# A case of intravascular histiocytosis associated with septic arthritis of a nonnative joint

**DOI:** 10.1016/j.jdcr.2025.09.028

**Published:** 2025-10-10

**Authors:** Alexander Idrogo-Lam, Michael Gui, Daniel Bennett, Mackenzie Asel

**Affiliations:** a University of Wisconsin School of Medicine and Public Health, Madison, Wisconsin; b Department of Dermatology, University of Wisconsin School of Medicine and Public Health, Madison, Wisconsin

**Keywords:** dermatopathology, intralymphatic histiocytosis, intravascular histiocytosis, orthopedic implants, septic arthritis, vascular lesions

## Introduction

Intravascular histiocytosis (IVH) is a rare disorder first described in 1994 that manifests as reticular erythematous or violaceous plaques, often mimicking other vascular lesions.^1^, ^2^, ^3^ The pathogenesis of IVH remains unclear, but it has been associated with chronic inflammatory states, autoimmune conditions, malignancies, trauma, and surgeries.^4^, ^5^, ^6^, ^7^ Specifically, rheumatoid arthritis has been implicated in almost 40% of cases.^8^^,^^9^ Skin biopsies of IVH show dilated dermal vessels with intraluminal clumps of mononuclear inflammatory cells.^6^ These intraluminal cells express histocyte specific markers (CD68 and CD163).^6^^,^^7^ In this case report we present a case of IVH in a patient with history of bilateral knee arthroplasty complicated by chronic septic arthritis.

## Case presentation

A 64-year-old man with a 6 year history of *Enterococcal* endocarditis status post aortic valve replacement, complicated by *Enterococcal* septic arthritis of bilateral knee arthroplasty, also with lung adenocarcinoma in remission presented with 1-year history of a “bruise” behind his left knee that had been spreading. He reported pink to purple discoloration behind the left popliteal fossa, which had since spread gradually to the distal thigh and proximal calf and gradually darkened. This lesion was intermittently tender to palpation but was not associated with pruritus, draining or ulceration. The patient denied fevers, chills, swelling of the knee, or trauma to the area.

**Fig 1:**
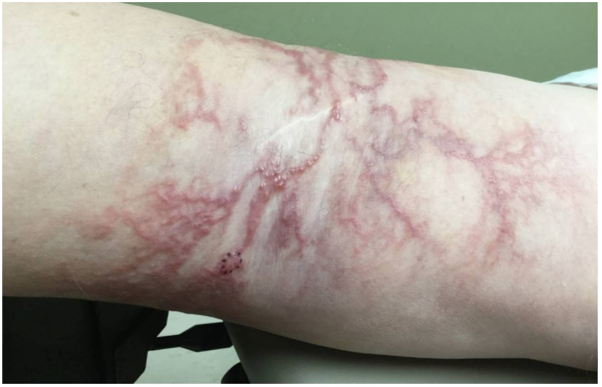
Image of lesion before biopsy at time of dermatology visit.

**Fig 2:**
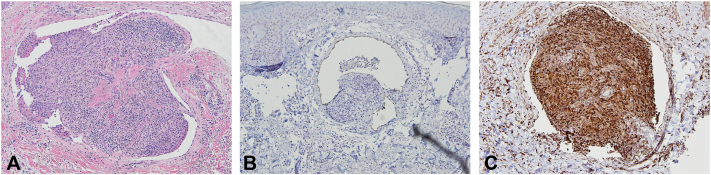
Skin biopsy of intravascular histiocytosis. **A,** Hematoxylin and eosin-stained sections demonstrating ectatic thin-walled vascular spaces containing a dense mixed inflammatory infiltrate with lymphocytes, plasma cells, and histiocytes associated with small thrombi. **B,** Immunohistochemical staining with D2-40 highlighting lymphatics containing histiocytes. **C,** Immunohistochemical staining with CD68 highlighting dense collections of histiocytes within vascular spaces. (Original magnifications: **A-C,** ×20.)

On physical examination, a well-demarcated, nonerythematous, reticulate rash with pseudovesiculation was noted on the left popliteal fossa, extending to the distal thigh and proximal calf (Fig 1). Pitting edema was noted in the left lower extremity. Given the unusual presentation and the patient’s surgical history, a 4 mm punch biopsy of the back of the left thigh was performed. The differential diagnosis for this lesion included lymphangioma, vasculitis, and surgical venous collateralization. Histology of the lesion demonstrated ectatic, thin-walled vascular spaces containing dense histiocytic infiltrates, accompanied by lymphocytes, plasma cells, and small thrombi (Fig 2, *A*). Immunohistochemical staining highlighted a D2-40^+^ lymphatic endothelium containing histiocytes (Fig 2, *B*), a mixed CD3 or CD20 lymphocytic population, some of which are within vascular spaces, and CD68^+^ intravascular histiocytes (Fig 2, *C*). These findings were most consistent with IVH. Additionally, there were features of papillary endothelial hyperplasia, suggesting a reactive process to thrombus formation and vascular occlusion.

## Discussion

Some consider IVH and intralymphatic histiocytosis (ILH) to be divided into 2 separate classifications: (1) with histocytes contained within lymphatics spaces (ILH), and (2) with histocytes within the intracapillary spaces (true IVH).^1^ Although there are additional clinical and histologic similarities between reactive angioendotheliomatosis and IVH, some believe ILH is a distinct clinical and histologic entity^1^^,^^3^ One study found that reactive angioendotheliomatosis and IVH were more associated with pain and thrombi compared with ILH.^1^ In our opinion, unequivocal distinction between ILH and true IVH may be limited given the small sample of most biopsies, perhaps not revealing the nature of all involved vessels.

The pathophysiology of IVH is not fully understood. Some reports suggest that IVH is a precursor to reactive angioendotheliomatosis.^1^ Others have suggested that IVH is a reactive phenomenon rather than a specific diagnosis.^5^^,^^6^ Chronic inflammatory responses to orthopedic implants may lead to histiocytic accumulation within vascular spaces. Repeated surgical trauma can cause scarring and impaired lymphatic drainage, promoting histiocytic sequestration.^4^^,^^10^ Additionally, vascular injury and subsequent thrombus formation may trigger a reparative response, as evidenced by papillary endothelial hyperplasia observed in this case. IVH represents a likely benign reactive condition that lacks clonal proliferation, unlike neoplasms such as Langerhans cell histiocytosis. Association with septic arthritis has not been previously described in the literature, but if the proposed reactive hypothesis is true, then it is reasonable that the septic arthritis may have promoted an inflammatory response and microtrauma that could have also contributed to this patient’s presentation. Although this patient had bilateral knee arthroplasty with septic arthritis, asymmetric differences in lymphatic drainage, inflammation, and scarring may explain the unilateral development of IVH. Additionally, even though the patient’s history of lung adenocarcinoma may have led to an increased inflammatory state, the absence of active tumor makes a paraneoplastic or systemic inflammatory mechanism less likely in this case.

Because of the low incidence of IVH, there is limited clinical recognition.^6^ Skin biopsy is necessary to help exclude other diagnoses and immunohistochemical stains including histocyte markers including CD68, CD163 are essential for diagnosis of IVH.^6^ A clear treatment for IVH has not been established. Reports of treatment have included, topical, intraarticular, or systemic corticosteroids, compression, antibiotics, and pentoxifylline, among others.^9^ There is limited evidence for a specific treatment and often there is spontaneous resolution without treatment.^3^^,^^6^ Revision of the underlying arthroplasty has also shown to resolve the lesion.^6^

Given that this is a benign and asymptomatic condition for the patient in this report, a conservative management approach was adopted. Compression therapy was initiated to improve vascular and lymphatic drainage. One year later the lesion persisted and topical steroids including triamcinolone acetonide 0.1% cream were used without notable improvement. The patient is currently seeking potential replacements of his knees owing to concerns for chronic infection.

## Conclusion

This case highlights the association between IVH and prior orthopedic interventions. The presence of papillary endothelial hyperplasia suggests IVH is a reactive condition due to the preceding trauma by his orthopedic implants. Chronic septic arthritis of his joint implants likely also triggered an inflammatory response and predisposed him to IVH. Clinicians should consider IVH in the differential diagnosis of persistent, noninfectious, noninflammatory cutaneous vascular lesions, especially in patients with a history of surgical procedures. A biopsy with immunohistochemical stains is essential for the diagnosis.

## Conflicts of interest

None disclosed.
